# Impact of a Novel PagR-like Transcriptional Regulator on Cereulide Toxin Synthesis in Emetic *Bacillus cereus*

**DOI:** 10.3390/ijms231911479

**Published:** 2022-09-29

**Authors:** Eva Maria Kalbhenn, Markus Kranzler, Agnieszka Gacek-Matthews, Gregor Grass, Timo D. Stark, Elrike Frenzel, Monika Ehling-Schulz

**Affiliations:** 1 Institute of Microbiology, Department of Pathobiology, University of Veterinary Medicine Vienna, Veterinärplatz 1, 1210 Vienna, Austria; 2 Department of Bacteriology and Toxinology, Bundeswehr Institute of Microbiology, Neuherbergstrasse 11, 80937 Munich, Germany; 3 Chair of Food Chemistry and Molecular Sensory Science, Technical University of Munich, Lise-Meitner-Straße 34, 85354 Freising, Germany

**Keywords:** *Bacillus cereus*, *pagR*, cereulide, transcription factor, homologue, *Bacillus anthracis*, toxins

## Abstract

The emetic type of foodborne disease caused by *Bacillus cereus* is produced by the small peptide toxin cereulide. The genetic locus encoding the Ces nonribosomal peptide synthetase (CesNRPS) multienzyme machinery is located on a 270 kb megaplasmid, designated pCER270, which shares its backbone with the *Bacillus anthracis* toxin plasmid pXO1. Although the *ces* genes are plasmid-borne, the chromosomally encoded pleiotropic transcriptional factors CodY and AbrB are key players in the control of *ces* transcription. Since these proteins only repress cereulide synthesis during earlier growth phases, other factors must be involved in the strict control of *ces* expression and its embedment in the bacterial life cycle. *In silico* genome analysis revealed that pCER270 carries a putative ArsR/SmtB family transcription factor showing high homology to PagR from *B. anthracis*. As PagR plays a crucial role in the regulation of the protective antigen gene *pagA*, which forms part of anthrax toxin, we used a gene-inactivation approach, combined with electrophoretic mobility shift assays and a bacterial two-hybrid system for dissecting the role of the PagR homologue PagRBc in the regulation of cereulide synthesis. Our results highlight that the plasmid-encoded transcriptional regulator PagRBc plays an important role in the complex and multilayered process of cereulide synthesis.

## 1. Introduction

*Bacillus cereus* is a major causative agent of two distinct forms of gastroenteritis diseases linked to food poisoning—emesis and diarrhea—as well as extra-intestinal infections, including endocarditis, endophthalmitis, and septicemia [1,2,3,4,5].

Most of the virulence factors of *B. cereus*, such as the diarrhea-causing enterotoxins, are located on the chromosome, while the *ces* genes – that are encoding the non-ribosomal peptide synthetase CesNRPS for the assembly of the emetic toxin cereulide—are located on a 270 kb plasmid pCER270. The megaplasmid pCER270 shares its origin of replication and its backbone with plasmid pXO1 from *Bacillus anthracis* [6,7]. The conserved state of pXO1 and pXO1-like plasmids is thought to reflect the relatively recent evolution of *B. anthracis* from a parental *B. cereus* subgroup [8]. The 182 kb pXO1 plasmid carries the anthrax toxin genes *cya*, *lef* and *pagA* as well as their major regulators *pagR* and *atxA* [2,9]. AtxA is known to act as the master virulence regulator in *B. anthracis*, exerting positive control on the anthrax toxin genes as well as the biosynthetic operon for the capsule synthesis, encoded on a second virulence plasmid pXO2 [10,11,12,13,14]. The weak autorepressor *pagR,* which is co-transcribed with *pagA*, acts in a complex signal-transduction cascade that controls the expression of virulence factors [15,16]. PagR exerts its negative control on *pagA* by directly binding to the *pagAR* promoter and is also involved in the transcriptional regulation of the chromosomally encoded S-layer genes *sap* and *eag* [15].

While considerable progress has been achieved in dissecting the regulatory circuits of anthrax toxin production in *B. anthracis*, much less is known about the regulation of cereulide toxin biosynthesis in emetic *B. cereus*. The *ces* locus consists of seven coding sequences (CDSs), the individually transcribed *cesH* and the polycistronically transcribed *cesPTABCD* gene cluster [17,18,19,20]. *cesH* encodes a putative hydrolase, *cesP* encodes a 4′-phosphopanthetheinyl transferase (PPTase) responsible for the activation of CesNRPS (nonribosomal peptide synthesis), *cesT* is a type-II thioesterase (TEII) with a proofreading function, *cesAB* encodes the structural cereulide synthetase genes and c*esCD* encodes an ABC transporter, recently shown to be directly involved in the tethering of the CesNRPS to the membrane [21]. The polycistronic transcription of *cesPTABCD*, which is driven by the main *ces* promoter P1, is strictly regulated and tightly linked to the physiological status of the cell [17,22,23,24].

In previous studies, knockout mutagenesis has shown that the transcriptional regulator PlcR, which is known to play a central role in the pathology of enterotoxigenic *B. cereus* [25], was not involved in the regulation of the emetic toxin cereulide [26]. However, the synthesis of cereulide is controlled by the Spo0A phosphorelay [26] and CodY [22]. In the early growth stages, the chromosomally encoded AbrB binds to the *cesP* promoter region and represses the *ces* transcription until *abrB* transcription is ceased by Spo0A [26]. Furthermore, CodY has been described to act as a master regulator interlinking synthesis of the emetic toxin and enterotoxins with the general metabolism [22]. However, since CodY, as well as AbrB, are only repressing cereulide synthesis during the earlier growth phases, other factors have to be involved in the strict control of *ces* expression during the bacterial life cycle. Indeed, it has been reported that CesH, which is part of the *ces* locus, is taking part in the transcriptional control of cereulide synthesis [20,27]. Since *cesH* transcription is upregulated at a later growth phase than the *ces* operon, CesH may contribute indirectly to the shutdown of *ces* mRNA synthesis after entering the stationary phase by degrading metabolites or signaling molecules. The direct action of CesH as a transcription factor is unlikely because it encodes for a bona fide hydrolase [27].

*In silico* search for putative transcription factors encoded on the megaplasmid pCER270 in emetic *B. cereus* revealed two ArsR/SmtB family members, showing high sequence similarity with PagR from *B. anthracis*. The ArsR/SmtB family is widely distributed throughout non-pathogenic and pathogenic bacteria including *Staphylococcus aureus, Pseudomonas aeruginosa* or *Mycobacterium tuberculosis* [28]. Members of the ArsR/SmtB protein family act primarily as prokaryotic transcriptional repressors, which regulate the expression of genes associated with metal(loid) sequestration or efflux in Gram-positive and Gram-negative bacteria [29,30]. Originally, ArsR/SmtB family members were described as metalloregulators and named after their founding members, the regulatory protein ArsR of the plasmid-encoded arsenical resistance operon in *Escherichia coli* and SmtB, which represses the transcription of a class II metallothionein gene *smtA* in *Synechococcus* PCC7942 [31,32]. Despite the original described function of ArsR/SmtB family in metal sensing and metal homeostasis, other ArsR/SmtB family members show a broad variety of regulatory functions, such as in symbiosis, biofilm formation, stress response and virulence [28]. For instance, ArsR/SmtB transcriptional regulators involved in the control of virulence factors have been described in *Mycobacterium tuberculosis* (Rv2034), *Vibrio cholera* (HlyU) and *B. anthracis* (PagR), [33,34,35]. Despite their diverse regulatory functionality, the ArsR/SmtB family shows a common tertiary structure consisting of five α-helices (α1-α5) and a typical hairpin flanked by the two anti-parallel β-sheets (β1 and β2), which enable homodimer formation [30].

In this study, we aimed at deciphering the potential role of the two PagR homologous ArsR/SmtB family transcriptional factors located on the pCER270 plasmid for cereulide bio-synthesis in emetic *B. cereus*. A gene-inactivation approach combined with electrophoretic mobility shift assays (EMSA) and a bacterial adenylate cyclase-based two-hybrid system (BACTH) allowed us to identify the first plasmid-encoded transcriptional regulator involved in the control of cereulide synthesis. This permitted us to gain further insights into the mechanisms orchestrating the interplay between chromosomally and plasmid-encoded factors controlling cereulide synthesis.

## 2. Results and Discussion

### 2.1. Identification of PagR-like ArsR/SmtB Family Regulators on the pCER270 Plasmid

**Figure 1:**
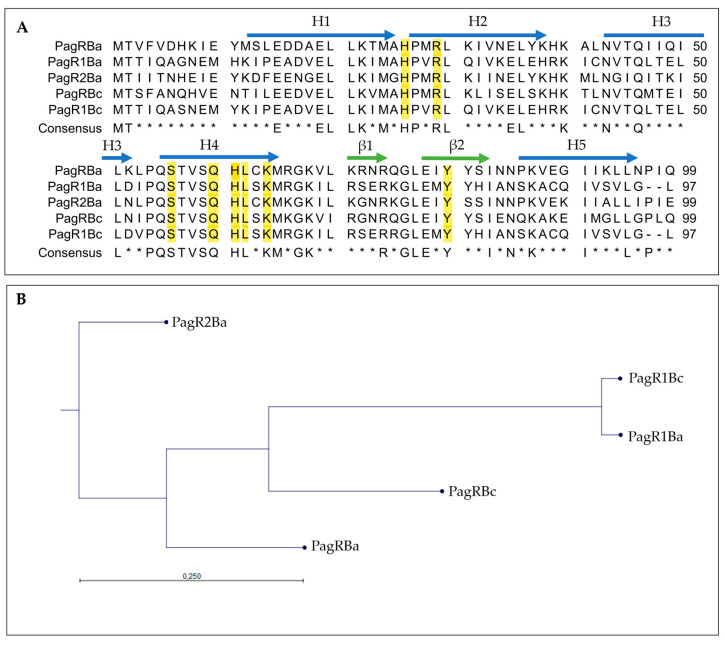
Amino acid sequence analysis of *B. anthracis* PagR (PagRBa) and its homologues on plasmids pXO1, pXO2 and pCER270. (**A**): Multiple sequence alignment of PagR homologues in emetic *B. cereus* and *B. anthracis*. The protein sequences of PagRBc (YP_002335935.1) from the emetic reference strain *B. cereus* F4810/72 and PagR1Bc (ACJ82764.1) were aligned to the two PagR protein sequences of *B. anthracis* Sterne encoded on pXO1 (PagR and PagR1) and the PagR homologue of *B. anthracis* Pasteur PagR2 encoded on pXO2 (for details of genome location, see Supplementary Figure S2). Sequences were retrieved from NCBI GenBank as follows: PagRBa (WP_000215715.1), PagR1Ba (AAD32441.1) and PagR2Ba (AJH31838.1). The consensus sequence is shown as identical amino acid residues in all PagRs in capital letters. Residues essential for DNA binding and physiological protein structure are highlighted in yellow [33]. Alpha helices (H1 to H5) are labelled with blue arrows and β-Sheets (β1 and β2) are labelled with green arrows. Asterisks * indicates amino acids not conserved in all sequences. (**B**): Protein similarity tree of the different PagR homologues based on the amino acid sequences of homologues from *B. cereus* (abbreviation: Bc) and *B. anthracis* (abbreviation: Ba) was calculated. The tree was constructed with the neighbor-joining method employing CLC Workbench Qiagen software. The Jukes–Cantor model was used for protein distance measurements.

We carried out an in silico analysis of the pCER270 sequence (GenBank accession number: DQ889676.1) to identify plasmid-encoded transcription factors potentially involved in the control of *ces* expression. We found two genes, BCAH187_RS28375 and BCAH187_RS28695, predicted to encode ArsR/SmtB family proteins, which we designated *pagRBc* and *pagR1Bc*, respectively. BCAH187_RS28375 and BCAH187_RS28695 are encoded on the sense strand, while des *ces* gene cluster *cesHPTABCD* is encoded on the anti-sense strand of pCER270 (see Supplementary Figure S1). The distance between the *ces* genes cluster and *pagRBc* is 102.5 kb, while *pagR1Bc* is located at a distance of 22 kb to the *ces* genes. Although *pagRBc* and *pagR1Bc* show sequence homology, there are no homologous genes found in their proximities (for details of the genetic *pagR* loci, see Supplementary Figure S2). However, a BlastP search revealed that PagRBc (YP_002335935.1) and PagR1Bc (ACJ82764.1) of emetic *B. cereus* show high similarity with *B. anthracis* PagR (PagRBa) and its homologues encoded on the *B. anthracis* virulence plasmids pXO1 (designated PagR1Ba) and pXO2 (designated PagR2Ba) (Figure 1A). The three-dimensional structure of PagR has been solved by multi-wavelength anomalous diffraction (MAD) and it was demonstrated that PagR bears the typical characteristics of an ArsR/SmtB family member, such as dimeric structure and a winged helix–turn–helix (HTH) DNA-binding domain, but lacks the classical metal-binding motifs [33]. A comparison of *B. anthracis* PagR and the PagR homologues of the emetic *B. cereus* and *B. anthracis* revealed that two specific protein motifs, “PQSTVSQHL” and “GLE”, are conserved in all PagR homologues (Figure 1A). The first motif represents the DNA recognition sequence α-helix α4 (also known as αR), ensures contact with the major groove of DNA and mediates protein–DNA interactions. The α4 is highly conserved and characteristic for the ArsR/SmtB family members [28]. Further, the three residues S56, Q60 and L62 in α4 of PagR are thought to interact with the DNA major groove [33]. The second motif, “GLE”, is located between the two β-sheets β1 and β2, forming a hairpin structure. Notably, the latter motif is also found in CadC, the transcriptional regulatory protein of the cadmium resistance system of *S. aureus* and *Listeria monocytogenes* [29]. *In silico* analysis of the protein sequences revealed that the residues, which are known to be crucial for DNA interactions of ArsR/SmtB repressors, are present in all PagR homologues (Figure 1A), indicating that these proteins are indeed ArsR/SmtB family members with yet to explore functionalities. With the exception of PagR2Ba, the residue Y81, which has been reported recently to be important for DNA binding of target gene promoters [36] and to be a potential tyrosine kinase phosphorylation site in PagR [16], is conserved in all PagR homologues. It is thus tempting to speculate that the PagR homologues share common molecular mechanisms.

Overall, PagR1Bc and PagR1Ba show the highest similarity among the PagR homologues, while PagR2Ba was found to be the most distantly related PagR homologue (Figure 1B). PagR1Bc and PagR1Ba differ only in four amino acid residues. Since these changes in the primary structure are located before or after the α-helices α1 to α5 and the β-sheets β 1 and β 2, it is tempting to speculate that both proteins are identical or very similar in their functional characteristics. The high similarity between PagR1Bc and PagR1Ba was also pinpointed by the results from a pairwise sequence alignment of PagR homologues performed by the Emboss Needle Algorithm to show an optimal sequence alignment consisting of identity, similarity, score and gaps (Supplementary Figure S3). This analysis revealed an identity of 95.9% and a similarity of 99.0% of PagR1Bc to PagR1Ba, while PagRBc and PagR1Bc only share an identity of 54.5% and a similarity of 73.7%. Interestingly, PagRBc showed the highest identity (63.6%) and similarity (78.8%) to PagRBa, which indicates that PagRBc might be a functional homologue of PagRBa.

**Figure 2:**
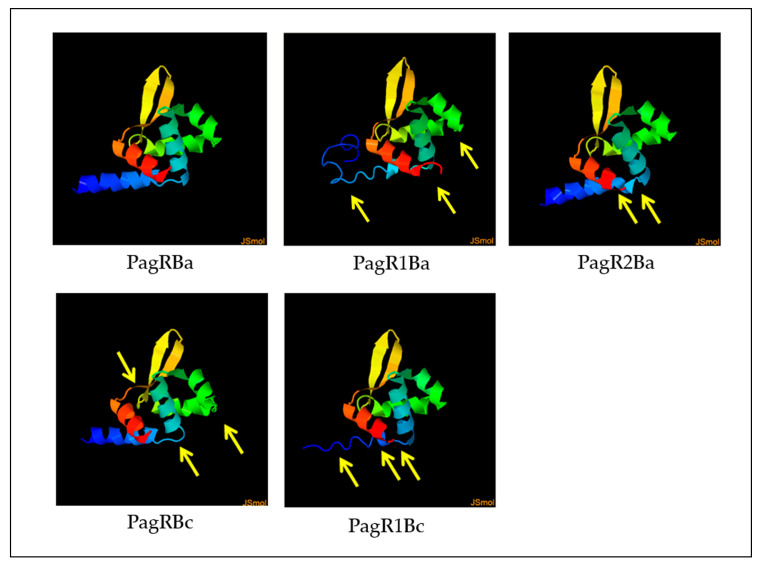
Predictive 3D protein models of PagR homologues. Structure models were constructed by the Phyre2 online tool, based on the crystal structure of PagR from *B. anthracis* [33]. Differences in protein folding are marked with yellow arrows of PagR homologues of emetic reference strain *B. cereus* F4810/72 and *B. anthracis* Sterne. Designations of PagR homologues as shown in Figure 1.

We next employed the Phyre2 online tool [37] to construct predictive 3D models of PagR homologues, based on the crystal protein structure and a 3D model of PagR (PagRBa) of *B. anthracis* [33]. As depicted in Figure 2, all PagR homologues show similar 3D structures resembling the typical architecture of ArsR/SmtB family transcription repressors, which leads to the unique structural folding of family members in the following order α1α2α3α4β1β2α5 [29,30]. However, while PagRBa, PagRBc and PagRBa2 are predicted to form all five α-helices (α1-α5) and the typical hairpin flanked by the two antiparallel β-sheets β1 and β2, the α1 helix seems to be only partially conserved in PagR1Ba and PagR1Bc.

### 2.2. In Vitro Binding of PagR Homologues to the ces Promoter Region

Since PagR is a DNA binding protein that exerts negative control of *pagAR* transcription in *B. anthracis* by direct binding to the *pagAR* promoter [16], we investigated the binding affinity of His_6_-tagged *B. cereus* PagR homologues (PagRBc, PagR1Bc) and the original His_6_-tagged PagR from *B. anthracis* (PagRBa) to the main promoter region of the *ces* operon in emetic *B. cereus*.

**Figure 3:**
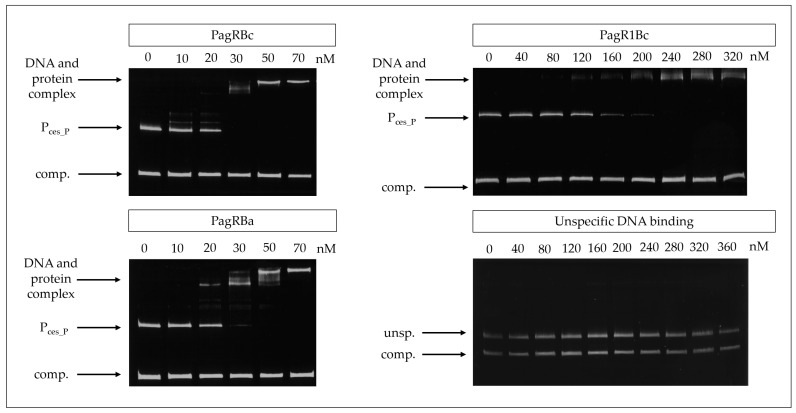
Gel mobility shift assay to determine the in vitro affinity of PagR homologues (PagRBc, PagR1Bc and PagRBa) to the *ces* gene promoter region of emetic *B. cereus*. The PagR homologues’ potential to bind to the *ces* promoter (P_ces_P_) was conducted in vitro using different amounts (0 nM up to 360 nM) of DNA comprising the promoter region of the *ces* operon or equimolar amounts of a competitive negative control DNA fragment (comp.), respectively. PagR concentrations are indicated in nM concerning the monomer on the top of each panel. Specificity of binding was further tested by using randomly amplified DNA from *B. cereus* (unsp.) with the competitive negative control DNA fragment (comp.) and increasing concentrations of purified PagR1Bc (lower right panel). A representative result from three independent experiments is shown.

For this, PagRBa, PagRBc, and PagR1Bc were heterologously expressed in *Escherichia coli* and the DNA-binding affinity of the purified proteins to the main *ces* promoter was tested by an electrophoretic mobility shift assay (EMSA). The promoter of the S-layer protein Eag, which is one of the main targets for PagR in *B. anthracis* [15], served as a positive control. Randomly amplified DNA fragments from emetic *B. cereus* F4810/72 served as a negative control. All three PagRs were able to bind the *cesP* probe containing the main *ces* promoter, while no specific interaction was observed within the unspecific DNA fragments (Figure 3). The highest binding affinity for the *ces* promoter was observed for PagRBc and PagRBa with an estimated equilibrium constant K_D_ of about 25 nM, whereas the binding of PagR1Bc was about six-fold weaker (K_D_ ≈ 150 nM). Notably, PagRBa and PagRBc showed a comparable binding affinity for the *eag* promoter of *B. anthracis* and emetic *B. cereus* (K_D_ of 20 to 25 nM), whereas the binding affinity of PagR1Bc to the *eag* promoter was much weaker (Supplementary Figure S4). Furthermore, our in vitro binding studies revealed that PagRBa as well as PagRBc formed two distinct protein–DNA complexes of different sizes, with an increase of the slower migrating complex at higher concentration of PagRBc and PagRBa, respectively (Figure 3 and Supplementary Figure S4). Previously, it has been reported that *S. aureus* CadC forms two protein–DNA complexes of different sizes. In line with our current findings, a shift to the higher molecular complex was observed with increasing amounts of CadC, possibly reflecting the binding of a second dimer to the DNA and the formation of a tetramer [38]. Although it has been reported that the binding of PagR dimers only covers 2.5 helical turns [33], it is known from DNase footprinting data that PagR binds to protect the DNA regions from the transcription at a length of nearly five helical turns [15]. Thus, it is tempting to speculate that PagR and its homologues may form tetrameric protein-DNA complexes to protect DNA regions from transcription *in vivo.* However, further studies, which are clearly beyond the scope of the current study, will be necessary to fully understand these highly dynamic and complex protein-DNA interactions.

Overall, our experiments demonstrate that PagRBc binds to the main promoter of the *ces* operon in vitro, suggesting that PagRBc may act as a transcriptional regulator of the cereulide synthetase gene cluster. Furthermore, the similar binding affinities of PagRBa and PagRBc for the *ces* and the *eag* promoter foster the hypothesis that these two proteins are not only structurally but also functionally homologues. Thus, we next invested their protein–protein interactions.

### 2.3. Interactions of PagR Homologues Identified by a Bacterial Two-Hybrid Assay

**Figure 4:**
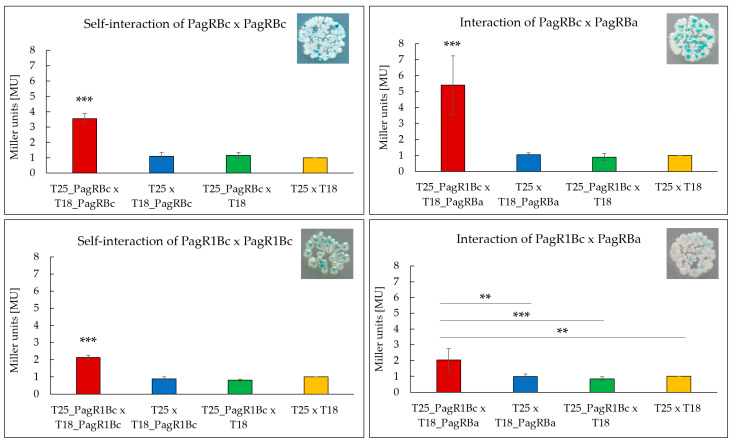
Screening of interacting PagR homologues of emetic *B. cereus* F4810/72 and *B. anthracis* Sterne strain by a Bacterial Two-Hybrid System Assay (BACTH assay). Self-interactions of the PagR proteins PagRBc and PagR1Bc are shown as well as the interactions of PagRBc × PagRBa and PagR1Bc × PagRBa. The efficacies of complementation between the indicated hybrid proteins were quantified by β-galactosidase assay (in Miller units [MU]). A color change to blue of the colonies indicates an interaction, respectively. Representative results from three independent experiments are shown, respectively. Statistically significant differences between the sample compared to the three controls are denoted as follows: ** *p* < 0.01, *** *p* < 0.001.

To test the ability of the *B. cereus* PagR homologues to form homodimers, which are the characteristic features of ArsR/SmtB family repressors [30], we used a bacterial adenylate cyclase-based two-hybrid system (BACTH) that allows testing protein interactions *in vivo* [39]. Both *B. cereus* PagR homologues were co-expressed with N-terminal and C-terminal fusions to the adenylate cyclase (*cya*) fragments T18 and T25, respectively, and tested for the formation of blue colonies on X-Gal/IPTG agar plates, thereby indicating protein interactions. To determine the affinity of the protein interactions, the enzymatic activity of β-galactosidase was additionally quantified in bacterial extracts by using the Miller Assay [40]. As depicted in Figure 4, the self-interaction of PagRBc was about 1.75-fold stronger than the self-interaction of PagR1Bc. The comparably weaker self-interaction of PagRBc might be explained by the lack of a folded α-helix α1 (Figure 2). Helices α1 and α5 have been reported to build the core of the binding surface for PagR dimers [33], which is crucial for the correct positioning of the α4 (=αR) helix for protein–DNA interaction.

In addition, we expressed *pagRBa* fused to the N-terminal *cya* fragment T18 together with each of the *B. cereus pagR* homologues fused to the C-terminal *cya* fragment T25 to investigate whether the predicted structural homologies are also reflected in functional interactions *in vivo*. As expected, the PagRBa showed stronger interactions with PagRBc (about 2.5-fold) than with PagR1Bc, reflecting once more the strong structural homologies between PagRBa and PagRBc. Since PagRBa is known to repress the expression of the anthrax toxin component *pagA*, we next tested the effect of PagRBc on the synthesis of the cereulide toxin by generating a *pagRBc* gene-deletion mutant.

### 2.4. Phenotypic Characterization of a pagRBc Gene-Deletion Mutant

**Figure 5:**
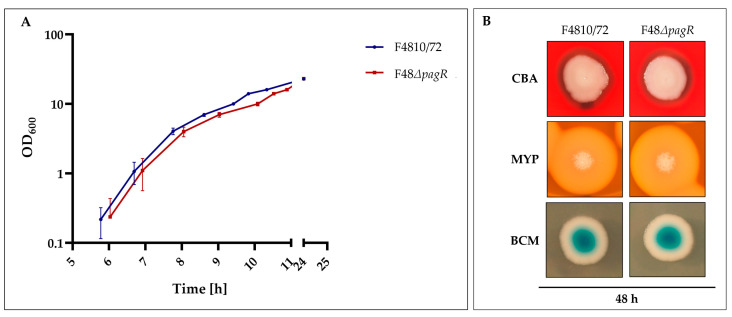
Growth and colony phenotypes of emetic *B. cereus* F4810/72 and its isogenic F48∆*pagR* mutant. (**A**): Growth curves of wild-type and F48*ΔpagR* mutant. Strains were cultivated in LB broth at 30 °C. The growth was monitored by measuring the optical density at 600 nm (OD_600_), shown are averages with standard deviations from three independent experiments. (**B**): Strains were grown on Columbia blood agar (CBA), Mannitol egg yolk polymyxin agar (MYP) and Brilliance *Bacillus cereus agar* (BCM), incubated at 30 °C for 48 h.

To assess the effect of PagRBc on cereulide biosynthesis, a single null mutant strain was constructed by allelic gene replacement of the *pagRBc* ORF in the emetic *B. cereus* reference strain F4810/72, resulting in the double-crossover *pagRBc*::*spc* strain F48*ΔpagR*. The growth of the *pagRBc* null mutant strain starts slightly later than the wild type but the increase in OD is similar (Figure 5A).

Furthermore, the F48*ΔpagR* and the wild-type strain F4810/72 did not exhibit differences in hemolysis nor in phosphatidylcholine- and phosphatidylinositol-specific phospholipases (PC-PLC and PI-PLC), as tested by growth at 30 °C on Columbia blood agar (CBA), Mannitol egg yolk polymyxin agar (MYP) and Brilliance *Bacillus cereus* agar (BCM), respectively (Figure 5B). These results suggest that PagR does not affect these chromosomally encoded virulence factors, which belong to the PlcR regulon [25].

### 2.5. PagRBc Acts as a Repressor of Cereulide Synthetase Gene Expression

**Figure 6:**
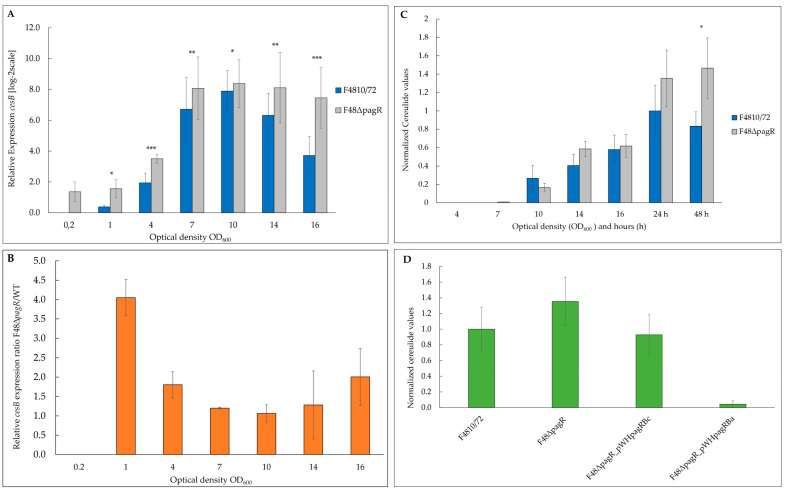
PagRBc represses cereulide toxin synthesis. The emetic reference strain *B. cereus* F4810/72 (wt) and its isogenic F48∆*pagR* mutant were grown in LB broth at 30 °C. Total RNA was extracted from *B. cereus* F4810/72 and F48∆*pagR*, harvested at indicated optical densities. Gene expression of *ces* and amounts of the cereulide toxin was determined at the OD_600_ as indicated. (**A**) The kinetics of *ces* transcription in F48∆*pagR* and its wild-type parent were determined by qRT-PCR. Levels of *ces* expression were determined by qRT-PCR and normalized to 16S *rrn* levels of the same sample preparations. The expression level of *ces* gene obtained for the wild-type strain at an OD_600_ of 0.2 (external calibrator sample) was set to 1 (R = 1) by default, and all other expression levels were compared relative to this condition using the REST method [41]. Data were calculated from at least two independent qPCR measurements from three independent biological replicates. Statistically significant differences between the strains are denoted as follows: * *p* < 0.01, ** *p* < 0.001, *** *p* < 0.0001. (**B**) Results of a comparative REST analysis of the relative *cesB* transcription levels of F48∆*pagR* and the wild-type strain. The *rrn*-normalized *cesB* expression values in F48∆*pagR* are depicted as log-2 ratios relative to the normalized *cesB* expression values of the wild-type strain at the same optical densities. Statistically significant differences in the *cesB* mRNA levels between the strains are indicated as denoted in (**A**). (**C**,**D**) The amount of cereulide in F48∆*pagR*, the wild-type strain and the *pagRBc* complementation strains was determined by UPLC-MS/MS. (**C**) Samples from F48∆*pagR* and the wild-type strain were analyzed at the OD_600_s and hours indicated. (**D**) In addition, cereulide was determined in F48∆*pagR* complemented *in trans* with *pagRBc* (F48*∆pagR*_pWH*pagRBc*) and *pagRBa* (F48*∆pagR_*pWH*pagRBa*), respectively; using a xylose-inducible promoter. Cereulide levels were calculated relative to the cereulide amount of the wild-type strain F4810/72 at 24 h. Samples were taken from three independent experiments shown as averages with standard deviations.

To examine the impact of PagRBc on *ces* transcription, we performed qRT-PCR-based quantification of mRNA levels. To compensate for slight growth delay of the mutant compared to its parental, we isolated RNA from cultures sampled at the same OD_600_ and monitored *ces* transcription from OD_600_ of 0.2 to 16 (Figure 6A). While the expression of *ces* in the wild-type strain was restricted to the late exponential/early stationary phase, the absence of *pagRBc* resulted in an approx. four-fold enhanced synthesis of *ces* mRNA during the early growth phase (Figure 6B). Generally, the *ces* mRNA levels in the *pagRBc* null mutant were significantly higher during the different growth phases, although both strains reached the highest *ces* transcription levels around the same OD_600_. In the wild-type *B. cereus* F4810/72, maximum *ces* transcription peaked at OD_600_ of 10 and declined thereafter, while the *ces* mRNA levels in the isogenic F48∆*pagR* mutant stayed constantly high. These results indicate that the pCER270 encoded *pagRBc* acts as a transcriptional repressor of the cereulide toxin synthetase operon *ces.*

In line with the results from the *ces* expression studies, the cereulide toxin was detectable earlier (at an OD_600_ of 7) by ultra-performance liquid chromatography (UPLC) tandem mass spectrometry (MS/MS) in the mutant F48∆*pagR* than in the *B. cereus* F4810/72 wild-type strain, and higher toxin levels were observed after 24 and 48 h (Figure 6C).

Since we found PagR (PagRBa) from *B. anthracis* not only to be structurally similar to *B. cereus* PagRBc but also to share functional characteristics with PagRBc, we examined if a heterologous PagRBa complementation strain could downregulate the increased cereulide production observed in the *pagRBc* null mutant. In parallel, we re-introduced *pagRBc* into F48∆*pagR* to generate a homologous complementation strain, in order to prove whether the increased cereulide production indeed would result from the deletion of *pagRBc*. Thus, we amplified *pagR* from *B. anthracis* Sterne and *pagRBc* from *B. cereus* F4810/72, respectively, fused the genes to a xylose-inducible promoter in the *B. cereus* expression vector pWH1520 and introduced these plasmid constructs into the F48∆*pagR* mutant. As expected, the induction of *pagRBc* with 0.1% xylose in the new strain—designated F48*∆pagR*_pWH*pagRBc*—reduced the cereulide levels to those observed in the wild-type F4810/72 strain. Furthermore, the induction of *pagRBa* with 0.1% xylose in the *pagRBa* complemented mutant strain—designated F48*∆pagR*_pWHpagRBa—led to a strong reduction of cereulide levels (Figure 6D). These results demonstrate that PagR plays a significant role in the regulation of cereulide biosynthesis. Furthermore, they show that besides *B. cereus* PagRBc, the structurally related PagR from *B. anthracis* substitutes the function of indigenous PagRBc in cereulide biosynthesis. The strong repressing effect of PagRBa on cereulide production observed in the heterologous complementation mutant indicates that PagR is able to form stable dimers, which mediate strong protein-DNA interactions.

Due to the high structural and functional homologies of PagRBa and PagRBc, we analyzed if the promoter regions of their target genes *eag* and *ces* comprise any conserved binding sites (see Supplementary Figure S5). However, as expected, no common binding site could be identified. Typically, ArsR/SmtB family members form dimeric or tetrameric proteins with a winged HTH-binding region and bind often imperfect inverted repeat motifs or imperfect palindromes [28,30]. Indeed, PagR (PagRBa) binds to DNA regions either symmetrically (to the *sap* promoter) or asymmetrically (to *pagA* and *eag* promoters, respectively), [15]. Thus, it is tempting to speculate that the *pagR* homologues in *B. anthracis* as well as in *B. cereus* follow a similar logic for binding to specific DNA regions. Another intriguing question that requires attention is the evolutionary origin of the PagR homologues encoded on the virulence plasmids of *B. anthracis* and emetic *B. cereus*, respectively. Although *B. cereus* pCER270 and *B. anthracis* pXO1 share a common backbone [7] and *pagR1Ba* and *pagR1Bc* are flanked by some homologous genes, the genetic organization of *pagRBa* and *pagRBc* is different (Supplementary Figure S2). There are no homologous genes found in the flanking genetic regions of *pagRBa* and *pagRBc* except for a Tn3 family transposase and its recombinase, which might be remnants from an ancient transition event. In contrast to *pagRBa*, which is known to be bicistronically transcribed with *pagA* [16], *pagRBc* is transcribed monocistronically, independently of the gene at its 5‘-proximity, designated *hyp1* (see Supplementary Figure S6). Thus, further studies will be necessary to fully decipher the molecular functionality of PagRBc and its role in the virulence of emetic *B. cereus*.

## 3. Materials and Methods

### 3.1. Bacterial Strains and Culture Conditions

A list of all bacterial strains used in this study is provided in Table 1. If not indicated otherwise, *E. coli* strains were routinely grown in LB broth (LB) or on LB agar plates at 37 °C. The emetic reference strain *B. cereus* F4810/72 and its derivatives were grown at 30 °C in LB broth or on LB agar plates. For kinetic inoculation of the main cultures with a final inoculum of 10^3^ CFU/mL of 100 mL fresh LB broth in baffled flasks, the emetic reference strain *B. cereus* F4810/72 and its derivatives were pre-cultivated for 16 h, as described previously [23,42]. Optical densities at 600 nm (OD_600_) were recorded using a Biospectrometer Basic (Eppendorf, Hamburg, Germany). To remain within the linear absorption capacity of the instrument, samples exceeding an OD_600_ of 1 were measured as 1:10 dilutions in fresh growth medium and samples exceeding an OD_600_ of 10 were measured 1:100 diluted, respectively, and extrapolated. The following antibiotics were added to the media when necessary: ampicillin (120 µg/mL), kanamycin (50 µg/mL) or 5-aminolevulonic acid (5ALA), [stock concentration: 50 mg/mL, final concentration 50 µg/mL) for *E. coli*; chloramphenicol (5 µg/mL) or tetracycline (10 µg/mL) for *B. cereus*.

### 3.2. General Molecular Methods

DNA isolation, manipulation, transformation of *E. coli*, plasmid preparation, protein separation by SDS-PAGE and immunoblotting was carried out according to standard procedures [47,48]. For cloning purposes and generation of gel mobility assay samples as well as protein samples, genomic DNA of the emetic *B. cereus* F4810/72, and the *B. anthracis* Sterne strain served as a template. DNA amplification was carried out with Phusion High-Fidelity DNA Polymerase (Thermo Fisher, Waltham, MA, USA). The oligonucleotides (synthesized by Eurofins Ebersberg, Germany) used for plasmid construction and strain manipulation are listed in Supplementary Table S1. An overview of plasmids used is provided in Supplementary Table S2. All constructs were verified by restriction enzyme digest and Sanger DNA sequencing (LGC Genomics, GmbH, Berlin, Germany). *E. coli* strains were transformed by heat shock and *B. cereus* strains by electroporation as described previously [19]. Conjugation was carried out according to Thoma and Schober [43] as described below (see Section 3.7).

### 3.3. Construction of E. coli Protein Expression Strains for Electrophoretic Mobility-Shift Assay (EMSA)

For recombinant protein expression, DNA fragments encoding *pagRBc* and *pagR1Bc* were amplified from genomic DNA of emetic *B. cereus* F4810/72 by using the following primer pairs: PagRNco_F/PagRXho_R and PagR1Nde_F/PagR1Xho_R (Supplementary Table S1). DNA fragments encoding *pagR* of *B. anthracis* Sterne strain were amplified from genomic DNA using the primer pairs: PagRBaNco_F/PagRBaXho_R (Supplementary Table S1). Probes were designed to achieve N-terminal or C-terminal His_6_-tag. Start and Stop codons were integrated and/or replaced in/with the respective restriction site. The amplified, digested and purified fragments were cloned into the expression vector pET28b(+). Constructs were verified by PCR and Sanger DNA-sequencing using the primers pET28b_for and pET28b_rev. For protein overexpression, the plasmids were introduced into *E. coli* BL21 (DE3) by transformation.

### 3.4. Production and Purification of Heterologous Proteins PagRBc-His_6_, PagR1-His_6_ of Emetic B. cereus and PagR-His_6_ of B. anthracis

Proteins were produced with *E. coli* BL21 (DE3) as soluble, N-terminal or C-terminal His_6_-tag fusions as previously described [22]. Protein production was induced by adding 1 mM IPTG to exponentially growing cells with an OD_600_ of 0.6. After 3 h incubation, cells were harvested at 6000× *g*, at 4 °C, 10 min and washed twice with washing buffer A [50 mM Tris pH 7.5, 50 mM KCl, 1 mM DTT, 0.5 mM Pefabloc (Roth, Karlsruhe, Germany)]. For protein purification, cells were resuspended in 3 mL Ni-NTA Native Lysis Buffer [50 mM NaH_2_PO_4_, 300 mM NaCl, 10 mM Imidazole, pH 8] containing 5 mM Pefabloc (Roth, Karlsruhe, Germany) and 3 µL of Benzonase [20,000 units], (Thermo Fisher, USA). Cells were disrupted twice with a French press (1 kbar) and cellular debris was removed by two times 20 min centrifugation step at 15,700 rpm at 4 °C. The supernatant was loaded onto Ni-NTA affinity columns (Qiagen, Chatsworth, CA, USA) pre-equilibrated with lysis buffer. The column was washed with 10 column volumes (CV) of washing buffer [50 mM NaH_2_PO_4_, 300 mM NaCl, 20 mM imidazole, pH 8.0] and the protein was eluted in elution buffer [50 mM NaH_2_PO_4_, 300 mM NaCl, 250 mM imidazole, pH 8.0]. The eluted fractions were dialyzed with the 10-fold BS buffer [50 mM Tris-HCl pH 7.5, 50 mM KCl, 10 mM MgCl_2_, 0.5 mM Na_2_EDTA, pH 8.0, 10% glycerol] using ultrafiltration columns with a 10 kDa cut-off (Vivaspin 500 concentrators (Sartorius AG, Goettingen, Germany)). Protein purity was analyzed by SDS-PAGE and total protein concentrations were determined with Pierce BCA Protein Assay Kit (Thermo Fisher, MA, USA), using bovine serum albumin as a standard.

### 3.5. Gel Mobility Shift Assay

Affinities of His_6_-tagged transcription factors PagR, PagR1 of *B. cereus* F4810/72 and PagR of *B. anthracis* Sterne to the promoter region of the *ces* gene were analyzed with nonradioactive native PAGE in gel mobility shift assays [49]. To analyze the binding to the *ces* promoter, a 523 bp fragment covering the main promoter region of *cesPII* [17], the *ces* promoter was amplified with the primer pairs: cesPII_for and cesPII_rev (Supplementary Table S1). The binding reactions contained increasing amounts (0 ng up to 360 ng) of PagR-His_6_, PagR1-His_6_ of *B. cereus* F4810/72 and PagR-His_6_ of *B. anthracis* Sterne and the *cesPII* promoter fragment in binding buffer [50 mM Tris-HCl (pH 8.0), 750 mM KCl, 2.5 mM Na_2_EDTA (pH 8.0), 0.5% Triton X-100, 62.5% glycerin (*v*/*v*), 1 mM dithiothreitol (DTT)]. To monitor nonspecific binding, equimolar amounts of a randomly amplified 301 bp DNA fragment (EMSAunspec7), originating from the *cesH* coding sequence of *B. cereus* F4810/72, served as competing DNA as described previously [26]. To further test the specificity of DNA-binding, PagR1-His_6_ was incubated with EMSAunspec7 and a second randomly amplified 201 bp DNA fragment (EMSAunspec8), origination from the *cesP* coding sequence of *B. cereus* F4810/72. The binding reaction was carried out as described above. Samples were incubated at RT for 30 min before being loaded onto a 10% native polyacrylamide gel which was run in pre-chilled TBE buffer at 120 V for 3 h at 4 °C. Gels were stained in ethidium bromide solution and visualized by UV irradiation.

### 3.6. Bacterial Two-Hybrid System (BACTH) and β-Galactosidase Assay

The genes *pagR1Bc* (NCBI locus tag: BCAH187_RS28695) and *pagRBc* (NCBI locus tag: BCAH187_RS28375) of the emetic *B. cereus* F4810/72, located on the pCER270 megaplasmid, and the *pagRBa* gene (NCBI locus tag AW20_RS00175) of *B. anthracis* (located on the pXO1-plasmid) were amplified (Supplementary Table S1) and cloned into the pKT25, pKNT25, pUT18 and pUTC18C BACTH expression vectors (Cat no: EUK001, Euromedex, Souffelweyersheim, France), (Table 1). The cloning procedure of the BACTH and the β-galactosidase assay were performed as recently described [21]. The respective plasmids were introduced into *E. coli* BTH101 *cya*-host strain by heat-shock transformation. The procedure is based on the functional complementation of two subunits of the adenylate cyclase (Cya) T18 and T25 fused with the putatively interacting partners as previously described [39,50]. Each protein was tagged on the N- and C-terminus by both subunits of Cya (T18 and T25). For each putative interaction, all possible combinations were tested in order to assess homo- as well as heterodimers by cotransforming plasmid constructs into *E. coli* BTH101 cells and plating them on LB X-gal/IPTG agar and LB IPTG for ß-galactosidase assay. The cells were incubated for 24 h at 30 °C, followed by incubation for 20 h at RT and for 20 h at 18 °C. For the β-galactosidase assay, cells were harvested from the LB-IPTG plates and resuspended in 1 mL Z-buffer (10 mM KCl, 10 mM MgSO_4_, 0.27% β-mercaptoethanol, Na-phosphate butter with pH of 7). The cell densities at OD_600_ were adjusted to 0.4 to 0.7, cells were permeabilized using β-mercaptoethanol, SDS and chloroform. The enzymatic reaction was carried out at 28 °C and started by adding 4 mg/mL ortho-Nitrophenyl-β-galactoside-sodium-phosphate buffer. After the samples turned yellow, the reactions were stopped with 1 M Na_2_CO_3_. The ß-galactosidase assay was carried out according to [50] and values were expressed in Miller Units [MU].

### 3.7. Construction of the B. cereus pagRBc Null Mutant Strain F48ΔpagR

To construct a *pagRBc* deletion mutant of the emetic *B. cereus* reference strain F4810/72, DNA regions of approximately 1500 bp flanking the *pagRBc* gene (NCBI locus tag: BCAH187_RS28375) were amplified with the primer pairs: pagRFl1_F/pagRFl1_R and pagRFl2_F/pag2Fl2_R for the flanking regions of *pagRBc* gene from emetic *B. cereus* F4810/72 DNA (Supplementary Table S1). The fragments were digested with KpnI/SacI and XhoI/XbaI, respectively. The amplification of a chloramphenicol-resistance cassette (1200 bp) from plasmid pAD123 [51] was carried out with the primer pairs CmEcoRI_F/CmEcoRI_R. The remaining fragment and the pCR 2.1 TOPO plasmid were digested with EcoRI and ligated by heat-shock transformation in *E. coli* TOP10 cells to result in pCR 2.1 TOPO/Cm construct (control primers for the insert were M13-F/M13-R). Both flanking region fragments were cloned into the chloramphenicol cassette containing plasmid pCR 2.1 TOPO/Cm. The construct was cut (KpnI/XbaI) and cloned into the similarly digested conjugative suicide pAT113 plasmid [52]. This plasmid was introduced into a diparental mating system with *E. coli* ST18, which was used to replace *pagRBc* with the chloramphenicol cassette in emetic *B. cereus* F4810/72 as described previously [43]. In brief, the diparental mating system is performed with *E. coli* ST18 strain, a *hemA* deletion mutant defective in tetrapyrrole biosynthesis, where the *hemA* mutation can easily be complemented by the addition of 5-aminolevulinic acid (5-ALA). The counterselection of the mating system is carried out by standard media and optimal growth conditions for the recipient strains. The conjugation was performed with the emetic *B. cereus* wild-type strain F4810/72 to yield the null mutant, designated F48Δ*pagR.* Gene deletion and integration of the resistance cassette was confirmed by PCR and sequencing using the primer pairs: pagRK1_F/pagRK1_R and pagRK2_F/pagRK2_R.

### 3.8. Expression of pagR Homologues in the pagRBc Null Mutant F48ΔpagR

To complement the *pagRBc* null mutant with *pagR* homologues from *B. cereus* as well as with *pagR* from *B. anthracis*, the respective genes were amplified from DNA of *B. cereus* F4801/72 and *B. anthracis* Sterne using the primers listed in Table S1. and introduced into pWH1520 plasmid to obtain pWH::*pagRBc* and pWH::*pagRBa*, in which the expression of *pagR* homologues is under control of a xylose-inducible promoter [53]. The plasmids were passaged through the non-methylating *E. coli* strain INV 110 and introduced into F48Δ*pagR* by electroporation. The successful uptake of the plasmids was verified by by PCR and subsequent sequencing of the PCR fragments with the primer pairs pWH1520_F/pWH1520_R (Supplementary Table S1). To induce the expression of the *pagR* homologues in F48Δ*pagR*, the cultures were kinetically inoculated 10^3^ CFU/mL as described above and induced with D-xylose to a final concentration of 0.1% (*v*/*v*), respectively, harvested at 24 h for determination of cereulide toxin levels.

### 3.9. Transcriptional Analysis of cesB Expression by qRT-PCR

Transcription of the *cesB* gene in the *B. cereus pagRBc null mutant* F48Δ*pagR* and its parental strain was analyzed by qRT-PCR as described previously [23,42]. For RNA isolation, 2 mL to 5 mL bacterial culture was harvested (10,000× *g*, 4 °C, and 2 min) at different optical densities. The supernatant was discarded and the cell pellets were directly frozen using liquid nitrogen. Pellets were stored at −80 °C until RNA extraction. RNA was isolated from frozen cell pellets via TRIzol Reagent (Invitrogen, Thermo Fisher, USA) and homogenized with FastPrep^®^-24 Ribolyser of MP (2 times of 45 s, in between samples were chilled on ice for 30 s, speed 6.5) using 0.1 mm ZnSn beads in 2 mL screw-top tubes. Phase separation was carried out with chloroform and nucleic acid was precipitated with 75% ethanol. RNA concentration was measured by Nanodrop™ 2000 spectrophotometer (Thermo Fisher, USA). Samples were diluted in a ratio 1:10 or 1:100, respectively, to get 1 µg final volume for cDNA synthesis with iScript™ gDNA Clear Synthesis Kit (Bio-Rad Laboratories, Vienna, Austria). The cDNA was diluted in a ratio 1:25 for qRT-PCR performance. For qRT-PCR analysis, we used SSO Advanced Universal SYBR Green Supermix (Bio-Rad Laboratories, USA) according to manufacturer instructions. For each reaction, 10 µL of SYBR Green, 2.5 µL of primer_F (3 µM) and 2.5 µL primer_R (3 µM) was used. Primers for qRT-PCR are listed in Table S1. To every qRT-PCR reaction mix, 5 µL of 1:25 diluted cDNA was added (final volume of 20 µL). Reactions were run on Bio-Rad Cycler (Bio-Rad Laboratories, USA, CFX96 Real-Time System C1000 Touch). To monitor gene expression, the REST method (Relative Expression Software Tool) was used according to [41], the mathematical model for relative quantification in real-time PCR. As an internal calibrator, (with a relative expression value of 1.00) the *ces* gene expression at an OD_600_ of 0.2 was chosen. Sample-to-sample variation was corrected by using the 16S rDNA gene as a reference (*rrn*), [54]. Mean values and standard deviations were calculated from three independent experiments and two independent measurements. Statistically significant differences (*p* < 0.5, *p* < 0.01 and *p* < 0.001) compared to the wild-type reference strain *B. cereus* F4810/72 were calculated with a paired, two-tailed Student’s *t*-test.

### 3.10. Cereulide Quantification by Means of Ultraperformance Liquid Chromatography (UPLC) Tandem Mass Spectrometry (MS/MS)

Samples of 5 mL were taken from bacterial cultures at a specific OD_600_ of 4, 7, 10, 14, 16 as well as 24 h and 48 h and centrifuged at 8000× *g*, 2 min, 4 °C. Pellets were stored at −80 °C until further processing. For cereulide extraction, about 50 mg of bacterial mass was weighted by pipetting and re-suspended in the respective amount of ethanol absolute. The cereulide extraction was performed as previously described [42].

The mass spectrometric analysis was performed using a WatersXevo TQ-S mass spectrometer (Waters, Manchester, UK) combined with an Acquity UPLC i-class core system (Waters, Milford, MA, USA), as described previously [55]. The UPLC-MS/MS system was equipped with a 2.1 × 150 mm, 1.7μm, UPLC CSH C_18_ column (Waters, Manchester, UK). Measurements were executed in the positive electrospray ionization (ESI) mode as described previously [55]. The UPLC Xevo TQ-S system was operated with MassLynxTM 4.1 SCN 813 Software (Waters, Manchester, UK), and analysis and data processing were completed using TargetLynx (Waters, Manchester, UK). By means of the multiple reaction monitoring (MRM) mode, the ammonium adducts of cereulide (*m*/*z* 1170.7→qualifier: *m*/*z* 172.2, 314.2; quantifier: *m*/*z* 357.2) were analyzed for a duration of 25 ms. The analysis of MS-data was performed as described [56]. All samples were measured in two different dilutions as duplicates. Mean values and standard deviations were calculated from three independent experiments.

### 3.11. Sequence Analysis

For the emetic reference strain *B. cereus* F4810/72, the genomic information was retrieved from the NCBI website (GenBank accession numbers NC_011655.1 and CP001179.1) and used for homology search and analysis of the *pagR* sequence. For the reference *B. anthracis* Sterne strain (GenBank accession number CP009540.1) and for the reference *B. anthracis* Pasteur strain (GenBank accession number CP009475.1), the genome information was also retrieved from the NCBI website were used for homologue search and analysis of the *pagR* sequence.

The DNA and amino acid sequences were accessed from the NCBI database. Protein homology search was performed by using BLAST (http://blast.ncbi.nlm.nih.gov/Blast.cgi) and as well as PATRIC (https://patricbrc.org/), accessed on 6 August 2022. Multiple sequence alignments were carried out with CLC Workbench Qiagen Software and SnapGene Software (GSL Biotech, USA). The published annotations of the characteristic protein domains of the crystal structure of PagR of *B. anthracis* was used according to [33]. Phylogenetic tree analyses were performed with the construction method neighbor joining and the distance measurement with the Jukes–Cantor model, including bootstrap analysis via CLC Workbench Qiagen Software.

For sequence homology analysis of proteins, the database Emboss Needle program [57] was used (https://www.ebi.ac.uk/Tools/psa/emboss_needle/), accessed on 9 august 2022, due to an optimal sequence alignment, applying the Needleman-Wunsch algorithm.

Predictive 3D protein models were constructed using the Phyre2 online tool (http://www.sbg.bio.ic.ac.uk/phyre2/html/page.cgi?id=index), accessed on 29 august 2021, by adding the protein sequence, respectively [37].

## *4.* Conclusions

In summary, our work provides novel insights into the complex regulatory circuits governing the non-ribosomal biosynthesis of the depsipeptide toxin cereulide by the CesNRPS. As shown previously, different levels of regulation are involved in the tight control of cereulide production [24]. The chromosomally encoded global regulator CodY, which senses the physiological status of the cell and orchestrates virulence factor expression in emetic *B. cereus*, as well as the pleiotropic transition state regulator AbrB, plays an important role as repressors of *ces* transcription in early growth phases. Both have been shown to act on the timing of *ces* expression by direct binding to the central promoter *cesP* [22,26]. In addition, CesH, which forms part of the *ces* gene locus, has been shown to influence the *ces* expression, but most likely by an indirect mechanism [27] yet to be explored. Furthermore, the ABC transporter CesCD contributes to cereulide production through its recently described non-canonical function in CesNRPS assembly [21].

**Figure 7:**
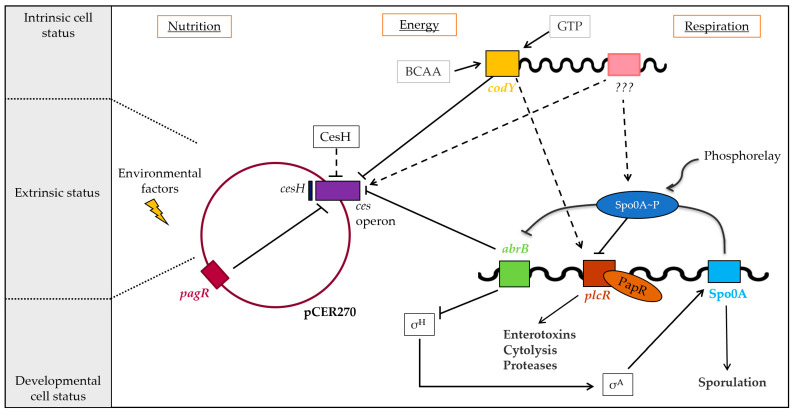
Regulation of cereulide toxin synthesis of emetic *B. cereus*. Cereulide toxin synthesis is a complex and multilayered process, orchestrated by the interplay of chromosomally and plasmid-encoded factors controlling the toxin synthesis at the transcriptional and post-transcriptional levels. The *ces* operon is tightly controlled by the chromosomally encoded global transcriptional regulators CodY and the transition phase regulator AbrB that belongs to the Spo0A regulon, but not by the pleiotropic *B. cereus* virulence regulator PlcR. The putative hydrolase CesH, encoded adjacent to the *ces* operon, indirectly controls cereulide biosynthesis, while the ArsR/SmtB family repressor PagRBc on pCER270 plasmid exerts control of *ces* transcription. Solid arrows: transcriptional regulation; dashed arrows: indirect regulatory effects (adapted from [24]).

With the identification of the PagR homologue PagRBc, which represents the first pCER270-encoded transcription factor described to be involved in *ces* transcription, we add a new piece to this still not entirely completed puzzle of virulence gene regulation in emetic *B. cereus* (Figure 7). Furthermore, PagRBc is the first ArsR/SmtB family member shown to be involved in the regulation of non-ribosomal assembly of cereulide by CesNRPS, highlighting the structural and functional diversity of factors involved in the tight control of *ces* expression. The homologous PagR (PagRBa) in *B. anthracis* has been shown to act in a complex cascade of signaling transduction that orchestrates the expression of virulence factors at the right time and in the right place [10,15,58]. It is therefore expected from our now-expanded understanding that PagRBc plays a similar role in the fine tuning of virulence factor expression in emetic *B. cereus*.

In addition, our work revealed that the newly identified PagR homologues PagR1Bc and PagR1Ba are nearly identical and also show a conserved genetic organization of their gene neighborhood, suggesting that they are functionally and structurally interchangeable. Notably, both genes are located on megaplasmids, which carry the key virulence determinants of emetic *B. cereus* (pCER270) and *B. anthracis* (pXO1 Ba), respectively. Thus, further studies should dissect the function of these PagR-like transcription factors for the pathophysiology of emetic *B. cereus* and *B. anthracis*.

## Figures and Tables

**Table 1:** Bacterial strains used in this study.

Strain or Plasmid	Relevant Genotype or Characteristics	Reference or Source
***E. coli***		
*E. coli TOP 10*	General cloning host	Invitrogen
*E. coli INV110*	Methylase-deficient cloning host	Invitrogen
*E. coli ST18*	*E. coli S17lpir* ∆*hemA*	[43]
*E. coli BL21(DE3)*	Protein expression strain	Novagen
*E. coli BL21* pET28b-*pagRBc*	Protein production strain for PagRBc used in EMSA	This study
*E. coli BL21* pET28b-*pagR1Bc*	Protein production strain for PagR1Bc used in EMSA	This study
*E. coli BL21* pET28b-*pagRBa*	Protein production strain for PagRBa used in EMSA	This study
*E. coli BTH101 cya*	Adenylate cyclase (*cya*) deficient reporter strain for bacterial two-hybrid screen (BACTH); F^−^, *cya*-99, *ara*D139, *gal*E15, *gal*K16, *rps*L1 (Str^r^), *hsd*R2, *mcr*A1, *mcr*B1	Euromedex
***B. anthracis***		
Sterne Strain	Vaccine strain devoid of pXO2 virulence plasmid	[44]
***B. cereus***		
F4810/72	Emetic reference strain, wild type; also termed AH187	[45,46]
F48*ΔpagR*	F4810/72 ∆*pagR::*cm; cm^r^	This study
F48*ΔpagR_pWHpagRBc*	F48Δ*pagR* harboring pWH::*pagR* of emetic *B. cereus* ^1^ for PagRBc production, Tc^r^	This study
F48*ΔpagR_pWHpagRBa*	F48Δ*pagR* harboring pWH::*pagR* of *B. anthracis* ^2^ for PagRBa production, Tc^r^	This study

^1^ Abbreviation of *B. cereus* is Bc. ^2^ Abbreviation of *B. anthracis* is Ba.

## Data Availability

Data are contained within the article or Supplementary Materials.

## Supplementary Materials

The following are available online at https://www.mdpi.com/article/10.3390/ijms231911479/s1.
