# Supplementary material for: Impact of a Novel PagR-like Transcriptional Regulator on Cereulide Toxin Synthesis in Emetic Bacillus cereus
Source: Int J Mol Sci. 2022 Sep 29;23(19):11479. doi: 10.3390/ijms231911479 (PMC9570423; doi:10.3390/ijms231911479)
Supplement: Supplementary file 1 [file ijms-23-11479-s001.zip › ijms-1885680-supplementary.pdf]

International Journal of *Molecular Sciences*

MDPI

Supplement

Eva Maria Kalbhenn [1], Markus Kranzler [1], Agnieszka Gacek-Matthews [1,†], Gregor Grass [2], Timo D. Stark [3], Elrike Frenzel [1,††], and Monika Ehling-Schulz [1,*]

[1] Institute of Microbiology, Department Pathobiology, University of Veterinary Medicine Vienna, Veterinärplatz 1, 1210 Vienna, Austria
[2] Department of Bacteriology and Toxinology, Bundeswehr Institute of Microbiology, Neuherbergstrasse 11, 80937 Munich, Germany
[3] Chair of Food Chemistry and Molecular Sensory Science, Technical University of Munich, Lise-Meitner-Straße 34, 85354 Freising, Germany
* Correspondence: monika.ehling-schulz@vetmeduni.ac.at (M.E.S.)
† Current address: Institute of Molecular Biotechnology of the Austrian Academy of Sciences (IMBA), Vienna BioCenter (VBC), Dr. Bohr-Gasse 3, 1030 Vienna, Austria.
†† Current address: Dr. Brill + KEBOS GmbH & Co.KG—Institute for Hygiene and Microbiology, Grützmühlenweg 48, 22339 Hamburg, Germany

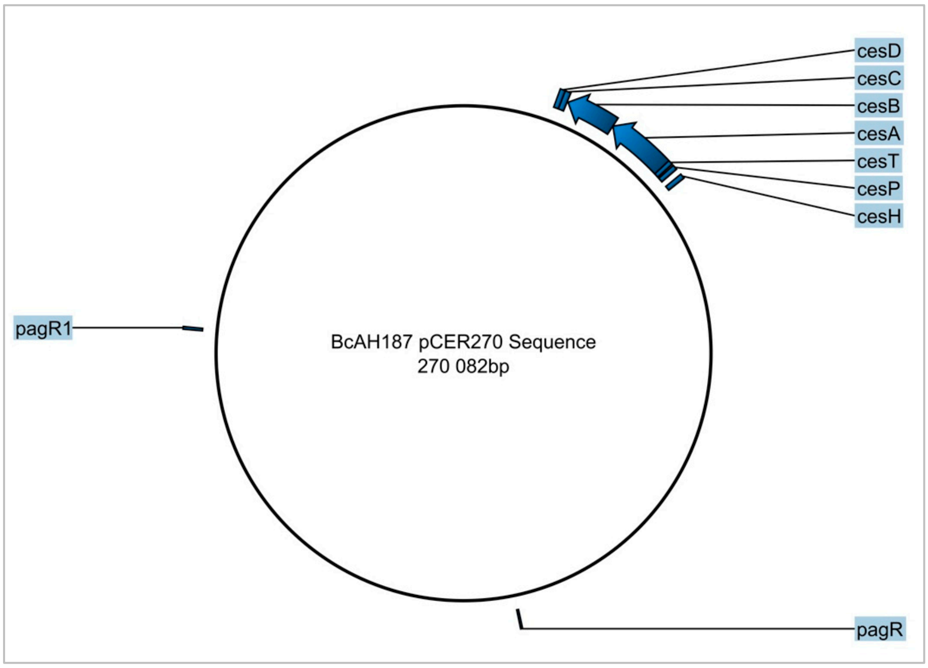

Figure S1. Location of *pagR* homologs on pCER270 of emetic *B. cereus*. Plasmid map of the pCER270 megaplasmid of the emetic refernce sterain F4810/72 (also known as AH187) depicting the genomic location of BCAH187_RS28375 and BCAH187_RS28695, predicted to encode ArsR/SmtB family proteins. Due to their homology to the transcriptional regulator *pagR* (see figure 1), encoded on the pXO1 toxin plasmid in *B. anthracis*, they have been designated as *pagR* and *pagR1* respectively. In addition, the *ces* gene, encoding the non ribosomal peptide synthetase CesNRPS responsible for bioysnthesis of the cereuldie toxin [20], is indicated. The plasmid map was generated with the CLC Workbench Qiagen Software.

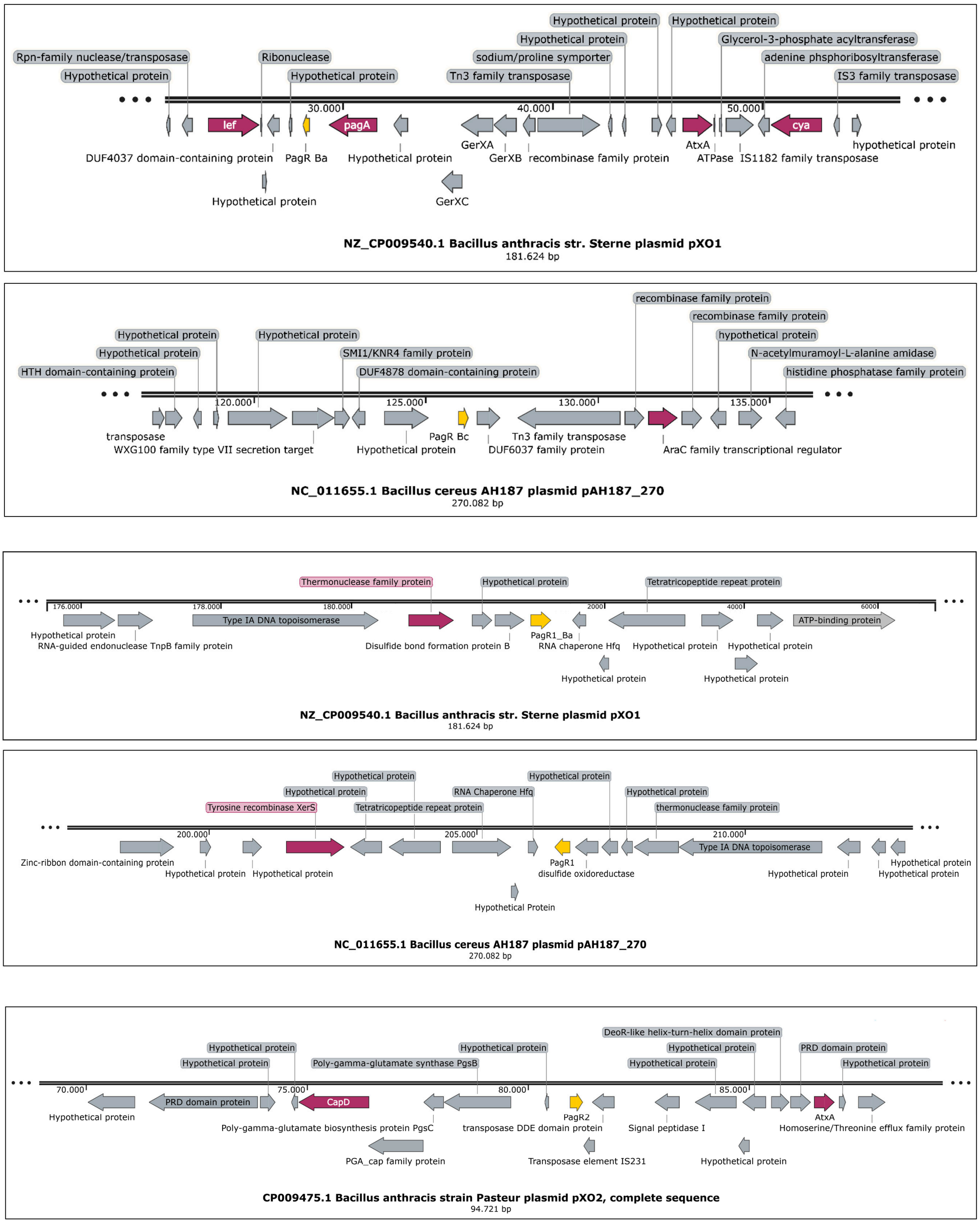

Figure S2. Genetic localization of the *pagR* homologues of emetic *B. cereus* F4810/72 (NC_011655.1) and *B. anthracis* Sterne strain pXO1 plasmid (CP009540.1), Pasteur Strain pXO2 plasmid (CP009475.1). Genes/Proteins are labelled with arrows based on their orientation on their plasmid. The *pagR* genes from *B. cereus* and *B. anthracis* are marked with a yellow arrow. Construction was performed with SnapGene Software (GSL Biotech, USA).

		**emetic *B.cereus***								***B. anthracis***											
		**PagR**				**PagR1**				**PagR**				**PagR1**				**PagR2**			
**emetic *B.cereus***	**PagR**	100%	100%	493	0%	54.5%	73.7%	279	2%	63.6%	78.8%	327	0%	55.6%	74.7%	281	2%	60.6%	77.8%	309	0%
	**PagR1**					100%	100%	485	0%	51.5%	63.6%	237	2%	95.9%	99.0%	472	0%	49.5%	67.7%	225	2%
B. anthracis	**PagR**									100%	100%	503	0%	51.5%	63.1%	236	9.7%	70.7%	81.8%	363	0%
	**PagR1**													100%	100%	488	0%	49.5%	67.7%	223	2%
	**PagR2**																	100%	100%	506	0%

Figure S3. Protein sequence homology of all PagR homologues of emetic *B. cereus* and *B. anthracis* strains. Identity (1st value), Similarity (2nd value), score (3rd value) and gaps (4th value) are illustrated. This analysis was based on EMBOSS Needle algorithm, the pairwise sequence alignment of proteins. The NCBI locus tag in *B. cereus* for PagR1Bc is BCAH187_RS28695, for PagRBc is BCAH187_RS28375. For *B. anthracis*, the NCBI locus tag is for PagRBa AW20_RS00175 [Sterne Strain], GBAA_RS29115 [Ames Ancestor], for PagR1Ba: AW20_RS00020 [Sterne Strain] and GBAA_RS29270 [Ames Ancestor]; for PagR2_Ba, the NCBI locus tag is BF26_RS00405 [Pasteur Strain] and GBAA_RS28255 [Ames Ancestor].

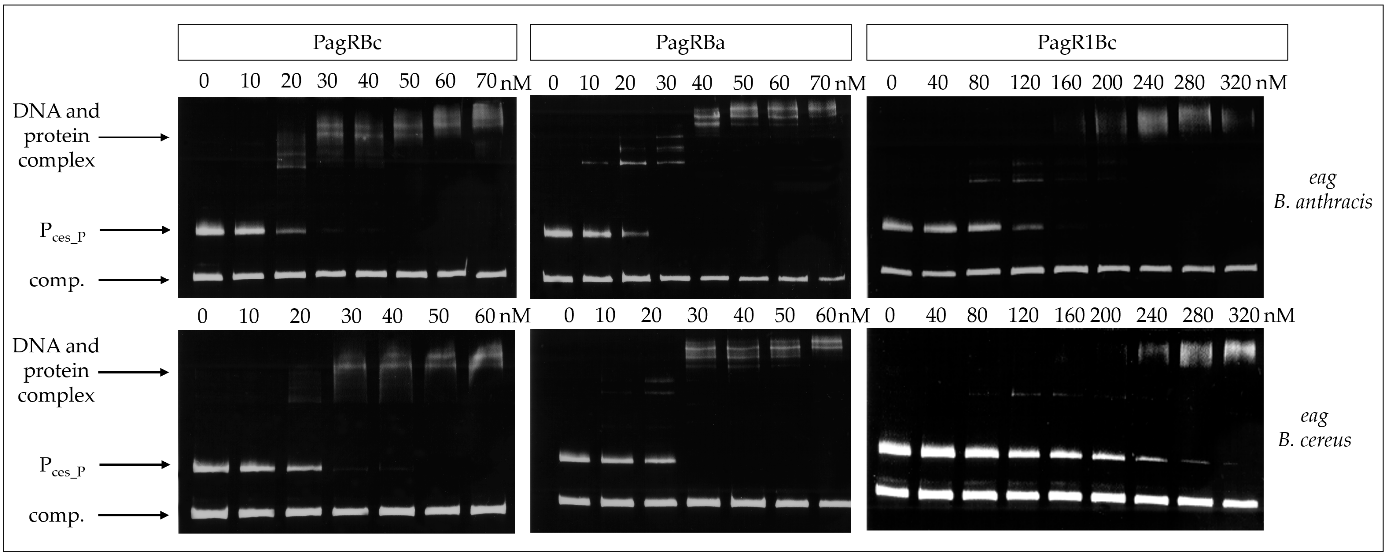

Figure S4. Gel mobility shift assay to determine the *in vitro* affinity of PagR homologs (PagR1Bc, PagRBc and PagRBa) to the *eag* gene promoter region of *B. cereus* and *B. anthracis*. The promoter of the S-layer protein Eag, which is one of the main targets for PagR in *B. anthracis*, served as positive control for the EMSA experiments. Similar to PagR from *B. anthracis*, binding of PagR homologs to the *eag* promoter of *B. anthracis* (*eag* Ba) and emetic *B. cereus* F4810/72 (*eag* Bc) was observed *in vitro* using different amounts of DNA comprising the promoter region of the *ces* operon and equimolar amounts of a competitive negative control DNA fragment (comp.), respectively. Note: PagR from *B. anthracis* and PagR from *B. cereus* showed a comparable binding affinity for the *eag* promoter of *B. anthracis* and emetic *B. cereus*, while the binding affinity of PagR1 was much lower. A representative result from three independent experiments is shown.

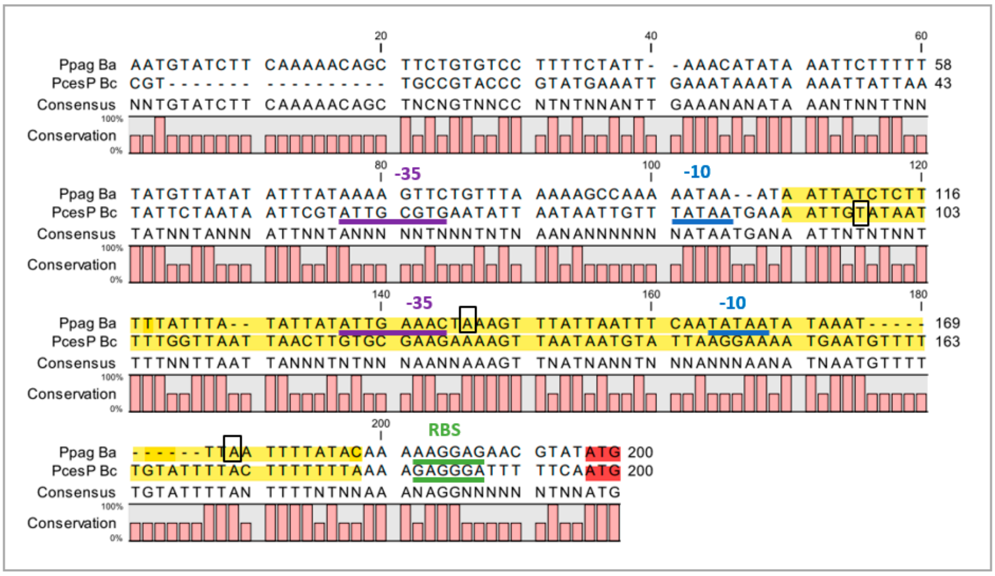

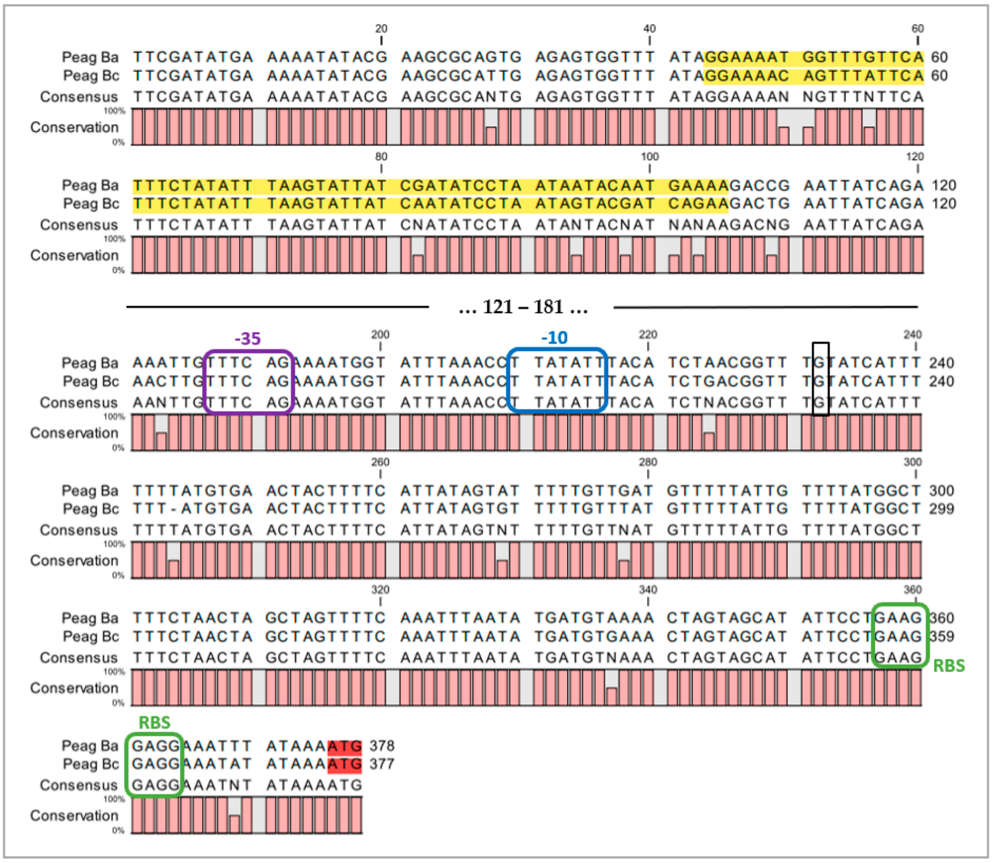

Figure S5. Alignment of the promoter regions P_{cesP} (Bc) and P_{pag} (Ba), as well as the regions of P_{eag} (Bc) and P_{eag} (Ba) are shown. Transcription start sites (+1) are boxed in black. Putative ribosome binding site (RBS) and putative -10 and -35 recognition sites are indicated in colored boxes. Translation start sites are marked in red. The *pagR* binding sites are marked in yellow [15].

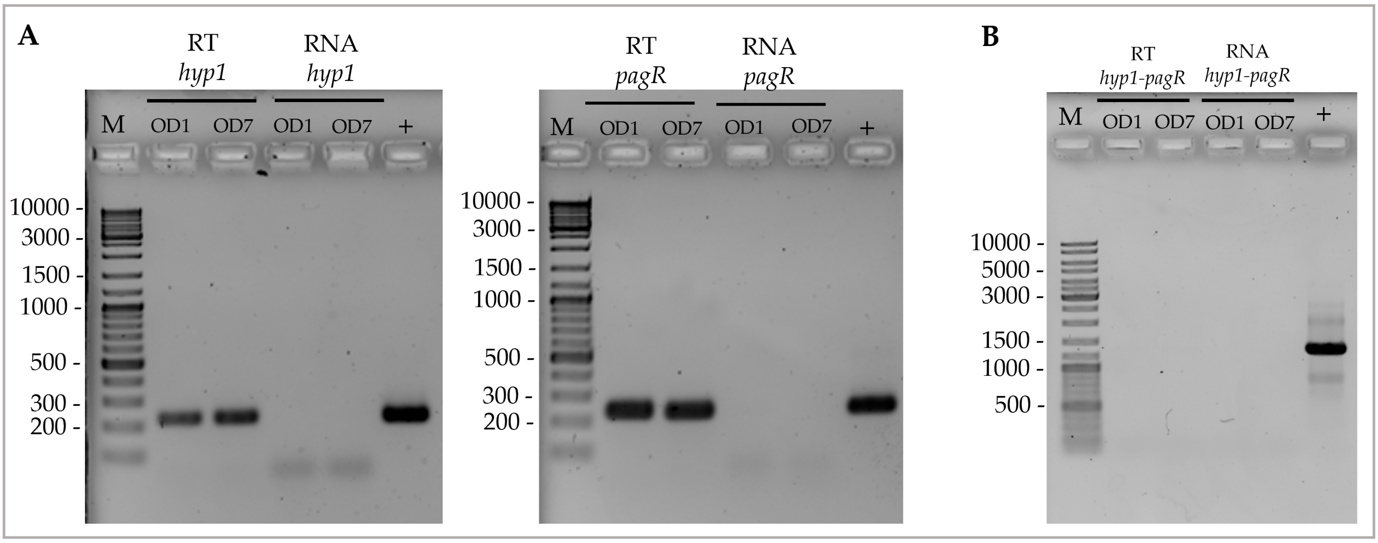

Figure S6. Test of transcription of *pagRBc* and the hypothetical gene, designated *hyp1*, in the 5′ proximity of *pagRBc.* RT-PCR, using internal primers of *hyp1* (qhyp1_F / qhyp1_R) and *pagRBc* (qpagR_F / qpagR_R), showed that both genes are transcribed at OD_{600} of 1 and 7 **(A)**. However, there were no transcripts from a forward primer in *hyp1* and reverse primer in *pagRBc* (qhyp1F / qpagR_R), indicating that both genes are independently transcribed **(B).** Negative controls (RNA, -) and positive controls (gDNA, +). M: marker ladder mixture (O′GeneRuler DNA Ladder Mix).

Table S1. Oligonucleotides used in this study. The sequence of the restriction enzymes is underlined, respectively.

Primer designation	Primer sequence (5′ → 3′)	Reference
**Construction of *E. coli* His6-tag protein overexpression strains**		
pET28b_for	TTAATACGACTCACTATAGGGG	Novagen
pET28b_rev	GCTAGTTATTGCTCAGCGG	Novagen
PagRBcNco_F	GTT<u>CCATGG</u>CTATGACAAGTTTTGCGA	This study
PagRBcXho_R	TT<u>CTCGAG</u>TTGTAACGGTCCTAATAAC	This study
PagR1BcNde_F	CTC<u>CATATG</u>ACAACTATTCAAGCAAGTAATGA	This study
PagR1BcXho_R	CAC<u>CTCGAG</u>GCTATTTAATTTGAGGTATTT	This study
PagRBaNco_F	GCT<u>CCATGG</u>CTATGACAGTATTTGTAG	This study
PagRBaXho_R	TG<u>CTCGAG</u>TTGGATAGGGTTTAACAAC	This study
**EMSA studies**		
cesPII_for (size 523 bp)	CTTCTTCAACGTGTGCTTCTA	[22]
cesPII_rev (size 523 bp)	GTGTTTCATTGAAAAATCCCTC	[22]
EMSAunsp7_for (size 301 bp)	ATGGTGGCGGAGTAAGTGGTTGGA	[22]
EMSAunsp7_rev (size 301 bp)	AAAGGAATCGGTTTAACCAACGCACTG	[22]
EMSAunsp8_for (size 201 bp)	GAACATTATTTTGCGAGAACGAG	[22]
EMSAunsp8_rev (size 201 bp)	TATTGGCGTTCTTGTTCTGTGAA	[22]
**BACTH studies**		
PagR1Bc_384F_PstI	AATTT<u>CTGCAG</u>GATGACAACTATTCAAGCAAG	This study
PagR1Bc_385R_XbaI	AAATT<u>TCTAGA</u>GTTAAACCTAGTACACTAACAATC	This study
PagR1Bc_386F_PstI	AATTT<u>CTGCAG</u>GGATGACAACTATTCAAGCAAG	This study
PagRBc_387F_PstI	AATTT<u>CTGCAG</u>GATGACAAGTTTTGCGAATC	This study
PagRBc_388R_XbaI	AAATT<u>TCTAGA</u>GTTTGTAACGGTCCTAATAACC	This study
PagRBc_389F_PstI	AATTT<u>CTGCAG</u>GGATGACAAGTTTTGCGAATC	This study
PagRBa_390F_PstI	AATTT<u>CTGCAG</u>GATGACAGTATTTGTAGATCAT	This study
PagRBa_391R_XbaI	AAATT<u>TCTAGA</u>GTTTGGATAGGGTTTAACAAC	This study
PagRBa_392F_PstI	AATTT<u>CTGCAG</u>GGATGACAGTATTTGTAGATCAT	This study
**Construction of *pagR* single knockout mutant**		
CmEcoRI_F	G<u>GAATTC</u>GGTTTTATCTTCGAGGATGC	This study
CmEcoRI_R	G<u>GAATTC</u>CGGGGCAGGTTAGTGAC	This study
M13-F	GTAAAACGACGGCCAG	universal
M13-R	CAGGAAACAGCTATGAC	universal
pagRFl1Kpn_F	TGATT<u>GGTACC</u>CGGTATATGGGAACCTGAGC	This study

pagRFl1Sac_R	TGATTGAGCTCGCTCTACATCCTCTTCTAAAATC	This study
pagRFl2Xho_F	TGATTCTCGAGGGGAAGGTTATAAGAGGTAATCGTC	This study
pagRFl2Xba_R	TGATTTCTAGAGCATTTTCCTGACGGGAC	This study
pagRK1_F	GCAGGGTAGCTATGATTCCTGC	This study
pagRK1_R	CTCTCCGTCGCTATTGTAACCAG	This study
pagRK2_F	GTGATGGTTATCATGCAGGA	This study
pagRK2_R	GCTCATCTTTTGGTGTACGC	This study
**Construction of *PagR* homolog complemented strains**		
pWH1520_F	GTTCACTTAAATCAAAGGGG	This study
pWH1520_R	GTCGGATCAATTCATCGATA	This study
pWHpagRBc_BcuI-F	GAACTAGTATGACAAGTTTTGCGAATCA	This study
pWHpagRBc_PaeI-R	CAGCATGCAATATTTTATTGTAACGGTC	This study
pWHpagR1Bc_BcuI-F	GCACTAGTAAAATGACAACTATTCAAGCA	This study
pWHpagR1Bc_PaeI-R	TCGCATGCTTATAAACCTAGTACACTAAC	This study
pWHpagRBa_BcuI-F	TGATTACTAGTGCTATGACAGTATTTGTAGATCAT	This study
pWHpagRBa_PaeI-R	AATCAGCATGCGAGGTAATTATATAAAATCTATTGGATAGG	This study
**RT-PCR**		
16S A1	GGAGGAAGGTGGGGATGACG	[54]
16S A2	ATGGTGTGACGGGCGGTGTG	[54]
qcesB_F	TTAGATGGTATTCTTCACTTGGC	[17]
qcesB_R	TTGATACAAATCGCATTCTTATAACC	[17]
qhyp1_F	CATGTTTACAGTGTCATAGGAGATAC	This study
qhyp1_R	CAGTCATATAATCTCCATACATATTCC	This study
qpagR_F	GACAAGTTTTGCGAATCAACACGTAG	This study
qpagR_R	CCTTGACGATTACCTCTTATAACCTTCC	This study

Table S2. Plasmids used in this study.

Plasmid	Relevant genotype or characteristics	Reference or source
pCR 2.1 TOPO	General cloning vector, Amp^r, Kan^r	Invitrogen
pCR 2.1 TOPO/Cm	Cloning vector from Invitrogen with additional Chloramphenicol Cassette cm^r; amp^r, kan^r	This study
pAT113	Suicide vector carrying the origin of IncP plasmids RK2 in order to allow conjugational transfer to gram-positive bacteria by *E. coli* strains with IncP plasmids, such as *E. coli* pRK24, *ori*R pACYC184, *ori*T RK24, Tra^-, Mob^+, *att*TN*1545*, MCS pUC19, kan^r, erm^r	[52]
pAD123	*B. cereus* – *E. coli* shuttle vector containing *gfp*mut3A, amp^r, cm^r	[51]

pWH1520	*Bacillus* sp. expression vector, xylose inducible; amp^r, tc^r	[53]
pWH::*pagRBc*	Promoter less *pagR* of emetic *B. cereus*[1] in pWH1520, amp^r, tet^r	This study
pWH::*pagR1Bc*	Promoter less *pagR1* of emetic *B. cereus*[1] in pWH1520, amp^r, tet^r	This study
pWH::*pagRBa*	Promoter less *pagR* of *B. anthracis*[2] in pWH1520, amp^r, tet^r	This study
pET28b(+)	*E. coli* expression vector, T7 lac promoter, His_6 tag; kan^r	Novagen
pET28b::*pagR1Bc*	Promoter less *pagR1* of emetic *B. cereus*[1] with N-terminal His_6 tag in pET28b, kan^r	This study
pET28b-::*pagRBc*	Promoter less *pagR* of *B. cereus*[1] with C-terminal His_6 tag in pET28b, kan^r	This study
pET28b::*pagRBa*	Promoter less *pagR* of *B. anthracis*[2] with C-terminal His_6 tag in pET28b, kan^r	This study
pKT25	BACTH expression vectors, T25 fragment of *cya* expressed from lac promoter, MCS is inserted at the 3' end (N-terminus) of T25, kan^r	Euromedex Cat No: EUK001
pKNT25	BACTH expression vectors, T25 fragment of *cya* expressed from lac promoter, MCS is inserted at the 5' end (C-terminus) of T25, kan^r	Euromedex Cat No: EUK001
pUT18	BACTH expression vectors, T18 fragment of *cya* expressed from lac promoter, MCS is inserted at the 5' end (C-terminus) of T25, amp^r	Euromedex Cat No: EUK001
pUT18c	BACTH expression vectors, T18 fragment of *cya* expressed from lac promoter, MCS is inserted at the 3' end (N-terminus) of T25, amp^r	Euromedex Cat No: EUK001
pUT:*pagR*Bc	*pagR* emetic *B. cereus*[1] inserted in frame with T18 fragment of *cya*, amp^r	This study
pUT:*pagR1*Bc	*pagR1* of emetic *B. cereus*[1] inserted in frame with T18 fragment of *cya*, amp^r	This study
pUT::*pagR*Ba	*B. anthracis pagR* inserted in frame with T18 fragmentof *cya*, amp^r	This study
pUTC::*pagR*Bc	*pagR* emetic *B. cereus*[1] inserted in frame with T18 fragment of *cya*, amp^r	This study
pUTC_*pagR1*Bc	*pagR1* emetic *B. cereus*[1] inserted in frame with T18 fragment of *cya*, amp^r	This study
pUTC_*pagR*Ba	*B. anthracis pagR* inserted in frame with T18 fragment of *cya*, amp^r	This study
pKT::*pagR*Bc	*pagR* emetic *B. cereus*[1] inserted in frame with T25 fragment of *cya*, kan^r	This study
pKT::*pagR1*Bc	*pagR1* emetic *B. cereus*[1] inserted in frame with T25 fragment of *cya*, kan^r	This study
pKT::*pagR*Ba	*B. anthracis pagR* inserted in frame with T25 fragment of *cya*, kan^r	This study
pKNT::*pagR*Bc	*pagR* emetic *B. cereus*[1] inserted in frame with T25 fragment of *cya*, kan^r	This study
pKNT::*pagR1*Bc	*pagR1* emetic *B. cereus*[1] inserted in frame with T25 fragment of *cya*, kan^r	This study
pKNT::*pagR*Ba	*B. anthracis pagR* inserted in frame with T25 fragment of *cya*, kan^r	This study

[1] Abbreviation of *B. cereus* is Bc.
[2] Abbreviation of *B. anthracis* is Ba.
